# Using computer assisted image analysis to determine the optimal Ki67 threshold for predicting outcome of invasive breast cancer

**DOI:** 10.18632/oncotarget.24398

**Published:** 2018-02-05

**Authors:** Timothy Kwang Yong Tay, Aye Aye Thike, Nirmala Pathmanathan, Ana Richelia Jara-Lazaro, Jabed Iqbal, Adeline Shi Hui Sng, Heng Seow Ye, Jeffrey Chun Tatt Lim, Valerie Cui Yun Koh, Jane Sie Yong Tan, Joe Poh Sheng Yeong, Zi Long Chow, Hui Hua Li, Chee Leong Cheng, Puay Hoon Tan

**Affiliations:** ^1^ Department of Anatomical Pathology, Singapore General Hospital, Singapore; ^2^ Current affiliation: Westmead Breast Cancer Institute, Westmead Hospital, Westmead, NSW, Australia; ^3^ Current affiliation: Q^2^ Solutions – Central Laboratories, Singapore Science Park One, Singapore; ^4^ Division of Medicine, Singapore General Hospital, Singapore; ^5^ Division of Pathology, Singapore General Hospital, Singapore

**Keywords:** Ki67, breast cancer, computer assisted image analysis, prognosis

## Abstract

**Background:**

Ki67 positivity in invasive breast cancers has an inverse correlation with survival outcomes and serves as an immunohistochemical surrogate for molecular subtyping of breast cancer, particularly ER positive breast cancer. The optimal threshold of Ki67 in both settings, however, remains elusive. We use computer assisted image analysis (CAIA) to determine the optimal threshold for Ki67 in predicting survival outcomes and differentiating luminal B from luminal A breast cancers.

**Methods:**

Quantitative scoring of Ki67 on tissue microarray (TMA) sections of 440 invasive breast cancers was performed using Aperio ePathology ImmunoHistochemistry Nuclear Image Analysis algorithm, with TMA slides digitally scanned via Aperio ScanScope XT System.

**Results:**

On multivariate analysis, tumours with Ki67 ≥14% had an increased likelihood of recurrence (HR 1.941, p=0.021) and shorter overall survival (HR 2.201, p=0.016). Similar findings were observed in the subset of 343 ER positive breast cancers (HR 2.409, p=0.012 and HR 2.787, p=0.012 respectively). The value of Ki67 associated with ER+HER2-PR<20% tumours (Luminal B subtype) was found to be <17%.

**Conclusion:**

Using CAIA, we found optimal thresholds for Ki67 that predict a poorer prognosis and an association with the Luminal B subtype of breast cancer. Further investigation and validation of these thresholds are recommended.

## INTRODUCTION

Ki67 is a nuclear antigen expressed in proliferating cells. An antibody to Ki67 labels proliferating cells throughout the non-G_0_ phases of the cell cycle and can therefore be used as a marker of cell proliferation. In breast cancers, Ki67 positivity has been shown to have an inverse relationship with disease free survival (DFS) and overall survival (OS)[1–3]. It has also been proposed to be useful in differentiating Luminal A from Luminal B molecular subtypes of oestrogen receptor (ER) positive breast cancers, as Luminal B tumours were found to have higher proliferative activity. Such a discrimination is especially important in patients with ER positive, node negative breast cancers where the Luminal subtype may influence decisions regarding adjuvant systemic therapy. However, as proliferation measured by Ki67 is a continuous variable which ranges from 0 to 100%, what constitutes the threshold of proliferative fraction that can stratify ER positive cancers into luminal A and B subtypes remains uncertain. There is currently no universal agreement on the cut off value that distinguishes the two, with some proposing a value of 14% or more [4] and others favouring a higher threshold of 20% and above [5]. Similarly, the threshold of Ki67 that correlates with adverse DFS and OS varies from study to study [6–10]. The method of evaluating Ki67 differs across published reports, making the results hard to compare. This can be due to differences in the type of antibody or antigen retrieval method used during the pre-analytical phase [11] or differences in visual assessment methods during the analytical phase, including both visual estimation or individual cell counting methods which can be associated with interobserver variability. Computer assisted image analysis (CAIA) has been found to ameliorate this problem of interobserver variability in Ki67 immunohistochemical interpretation [12–14]. We sought to investigate if there is an optimal cut off value for Ki67 in predicting survival outcomes as well as differentiating luminal A from luminal B subtypes of invasive breast cancer using CAIA, in a cohort of 440 patients diagnosed with invasive breast cancer in 2012.

## RESULTS

The clinico-pathological features of all 440 patients are summarised in Table 1. The age of the patients ranged from 22 to 91 years and mean and median age was 57 years. Of the series, Chinese ethnicity predominated with 343 (78%) patients, Malay 39 (8.9%), Indian 14 (3.2%) and other ethnic groups 44 (10%) respectively. Mean and median tumour sizes were 32mm and 25mm with a range of 12mm to 142mm. Invasive ductal carcinoma (NOS) was the subtype observed in 385 (87.5%) patients with 55 (12.5%) patients showing other histological subtypes (Table 2). Lymphovascular invasion was seen in 157 (35.7%) tumours and 134 (30.5%) patients had positive axillary lymph nodes. ER positivity was observed in 343 (78%) tumours, progesterone receptor (PR) positivity in 264 (60%) and HER2 positivity in 99 (22.5%) tumours respectively.

**Table 1:** Clinicopathological parameters (N=440)

Clinicopathological parameters	Number (%)
**Age (years) (mean 57, median 53, range 22 to 91)**	
<median age	217 (49.3%)
≥median age	223 (50.7%)
**Ethnicity**	
Chinese	343 (78.0%)
Malay	39 (8.9%)
Indian	14 (3.2%)
Others	44 (10.0%)
**Tumour size (mm)(mean 32, median 25, range 12 to 142)**	
≤ 20	157 (35.7%)
> 20	278 (63.2%)
Not available	5 (1.1%)
**Histologic grade**	
1	53 (12.0%)
2	169 (38.4%)
3	201 (45.7%)
Not available	17 (3.9%)
**Histologic subtype**	
Invasive ductal carcinoma, NOS	385 (87.5%)
Other subtypes (refer to Table 2)	55 (12.5%)
**Lymphovascular invasion**	
Absent	283 (64.3%)
Present	157 (35.7%)
**Axillary lymph node status**	
No nodal metastasis	97 (22.0%)
Metastasis in lymph nodes	134 (30.5%)
Not sampled	209 (47.5%)
**Estrogen receptor**	
Negative	97 (22.0%)
Positive	343 (78.0%)
**Progesterone receptor**	
Negative	176 (40.0%)
Positive	264 (60.0%)
**HER2**	
Negative	215 (48.9%)
Positive	99 (22.5%)
Equivocal	126 (28.6%)
**Immunohistochemical profile of tumours**	
ER and/or PR (+) and HER2 (−)	278 (63.2%)
ER and/or PR (+) and HER2 (+)	67 (15.2%)
ER (−), PR (−) and HER2 (+)	31 (7.1%)
ER (−), PR (−) and HER2 (−)	64 (14.5%)

**Table 2:** Histologic subtypes of 440 invasive breast cancers

Histologic subtype	No. (%)
Invasive ductal carcinoma, NOS	385 (87.5)
Invasive lobular carcinoma	18 (4.1)
Mucinous carcinoma	16 (3.6)
Invasive ductal and lobular carcinoma	9 (2)
Medullary carcinoma	3 (0.7)
Tubular carcinoma	3 (0.7)
Metaplastic carcinoma	2 (0.5)
Papillary carcinoma	2 (0.5)
Adenoid cystic carcinoma	1 (0.2)
Invasive cribriform carcinoma	1 (0.2)

### Ki67 immunohistochemistry (IHC) vs Ki67 mRNA

**Figure 1:**
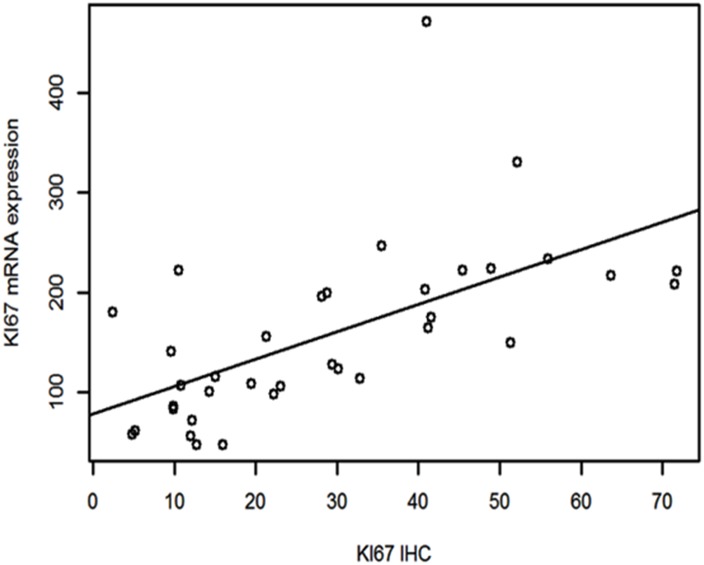
Linear regression between Ki67 mRNA expression and Ki67 IHC

Spearman’s correlation showed that there was a strong association between Ki67 mRNA expression and Ki67 IHC measured by CAIA with a correlation coefficient of 0.68 (p<0.0001). Linear regression (Figure 1) showed that Ki67 mRNA expression increased about 2.73 units (95% CI 1.54- 3.91) with every unit increase of Ki67 IHC (P=0.0001).

Of the total 1320 cores (440 × 3), only a small percentage (5.4%; 71 cores) were inadequate. Comparing the tumour cell count across all 3 cores using intraclass correlation coefficient (ICC) also revealed an ICC value of 0.645 on single measures and 0.784 on average measures which is moderate to strong agreement (Table 3). All 440 cases had at least 1 core that was adequate. In cases with two or three cores, the core which yielded the highest Ki67 proliferation rate was selected for analysis. Of these 440 TMA cores, mean and median tumour cell counts assessed by CAIA were 7,518 cells and 5,422 cells respectively with a range of 1,222 cells to 130,950 tumour cells. Table 4 shows the mean, median and range of Ki67 percentage immunoreactivity in the whole series including all invasive breast cancers and subsets of ER positive breast cancers. Mean Ki67 proliferation activity was 14% and 12% in the whole series and among ER positive cases respectively.

**Table 3A:** Intraclass Correlation Coefficient (ICC) value in comparing tumour cell count across 3 TMA cores

	Intraclass Correlation^a^	95% Confidence Interval		F Test with True Value 0			
		Lower Bound	Upper Bound	Value	df1	df2	Sig pvalue
Single Measures	**.645^b^**	.584	.700	4.640	390	390	**.000**
Average Measures	**.784^c^**	.737	.823	4.640	390	390	**.000**

**Table 3B:** Intraclass Correlation Coefficient (ICC) value in comparing tumour cell count between Aperio and Definiens system

	Intraclass Correlation^a^	95% Confidence Interval		F Test with True Value 0			
		Lower Bound	Upper Bound	Value	df1	df2	Sig p value
Single Measures	**.716^b^**	.498	.849	6.034	32	32	**.000**
Average Measures	**.834^c^**	.664	.918	6.034	32	32	**.000**

Two-way mixed effects model where people effects are random and measures effects are fixed.

a. Type C intraclass correlation coefficients using a consistency definition-the between-measure variance is excluded from the denominator variance.

b. The estimator is the same, whether the interaction effect is present or not.

c. This estimate is computed assuming the interaction effect is absent, because it is not estimable otherwise.

**Table 4:** Ki67 immunohistochemical expression in ER positive invasive breast cancer

	Number	Mean Ki67%	Median Ki67%	Range
Whole series	440	14.5023	10.882	0.39-71.77
ER+ cases	343 (78%)	12.0922	9.3281	0.39-57.11
**In ER+ series N=343**				
ER+HER2+ (68/343, 19.8%)	68 (19.8%)	12.2316	9.0678	0.80-57.11
ER+HER2- (169/343, 49.3%)	169 (49.3%)	12.3445	9.3248	0.40-52.12
ER+PR- (83/343, 24.2%)	83 (24.2%)	14.6159	10.8852	1.04-45.39
ER+PR+ (260/343, 75.8%)	260 (75.8%)	11.2264	8.9724	0.39-57.11
ER+PR<20% (130/343, 37.9%)	130 (37.9%)	13.8168	10.6703	0.53-52.12
ER+PR≥20% (213/343, 62.1%)	213 (62.1%)	11.0396	9.0152	0.39-57.11

### Aperio vs. Definiens image analysis platforms

Comparison of the Ki67 scoring by both image analysis platforms showed an intraclass correlation coefficient value of 0.731 (95% CI 0.573-0.836, p<0.001) and kappa value of 0.730 which is considered strong agreement (Table 5). In comparing the tumour cell count by the Aperio and Definiens system, we found an intraclass correlation coefficient value of 0.716 on single measures and 0.834 on average measures which is strong to almost perfect agreement (Table 3).

**Table 5:** Measure of agreement between Aperio and Definiens Image Analysis platforms

Aperio platform				
Definiens platform	Ki67<14%	Ki67≥14%	Total	
Ki67<14%	21 (84%)	4 (16%)	25	p<0.001
Ki67≥14%	3 (11%)	24 (89%)	27	kappa=0.730

### Survival analysis

**Figure 2:**
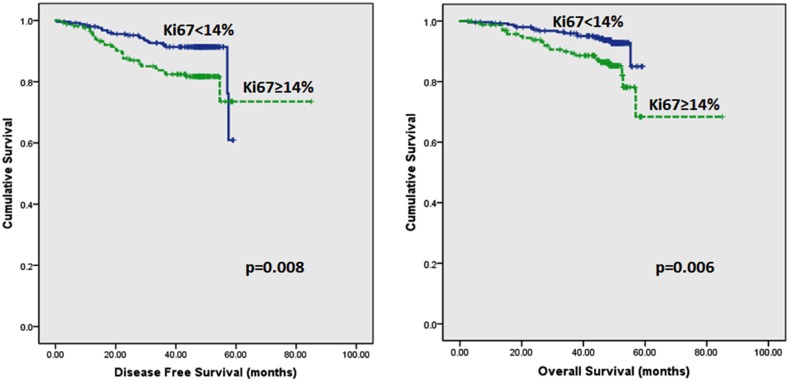
Kaplan-Meier disease free survival and overall survival curves of patients with tumours exhibiting Ki67<14% and ≥14% in all tumours

**Figure 3:**
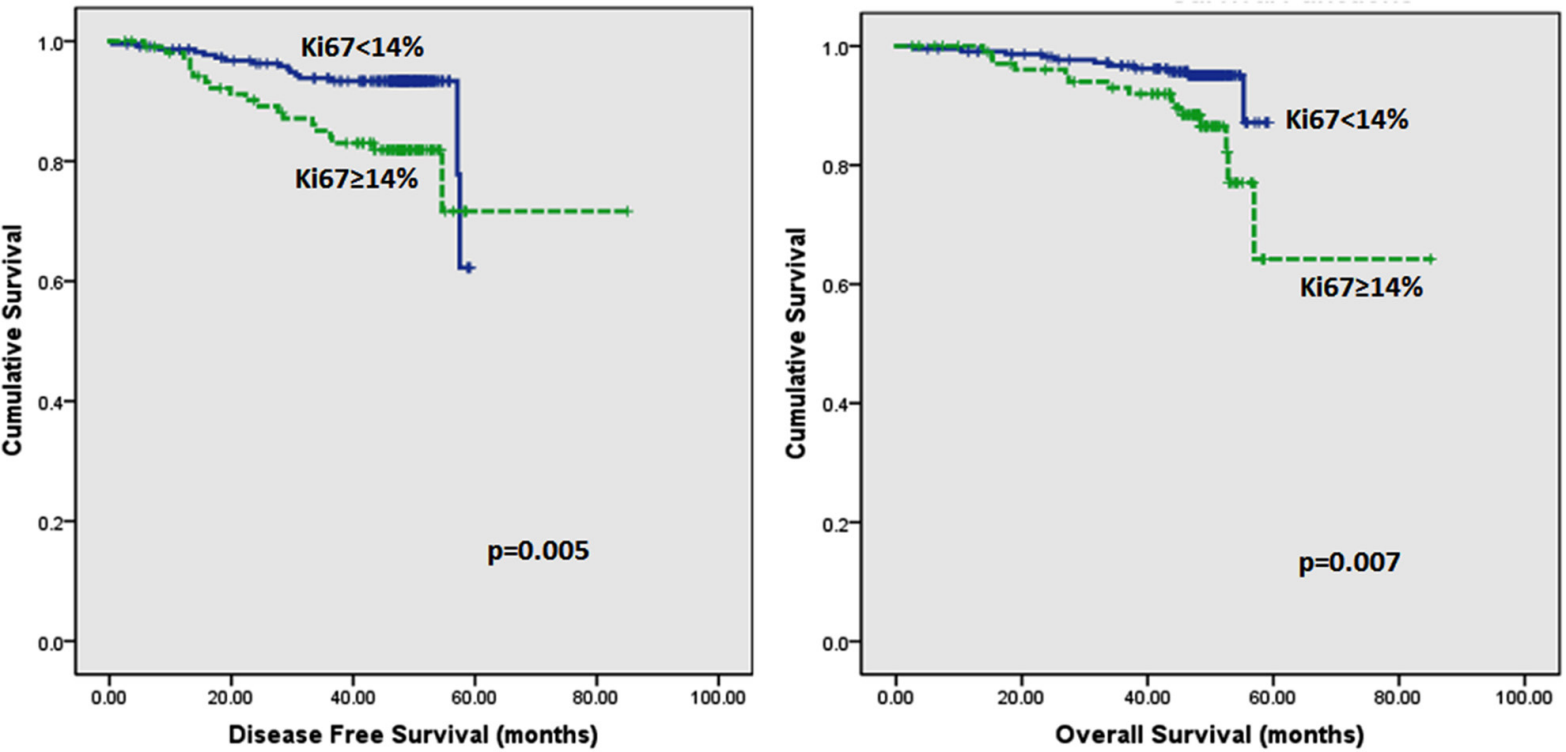
Kaplan-Meier disease free survival and overall survival curves of patients with tumours exhibiting Ki67 of <14% and ≥14% in ER positive tumours only

**Figure 4:**
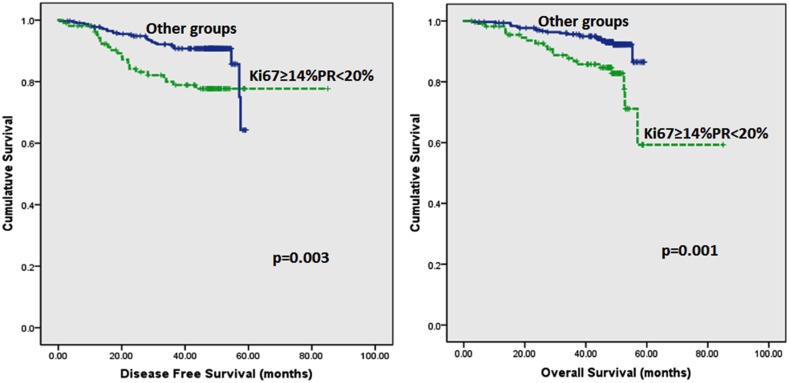
Kaplan-Meier disease free survival and overall survival curves of patients with tumours exhibiting an immunoprofile of Ki67≥14%PR<20% (Luminal B) compared with other immunoprofiles in all tumours

**Figure 5:**
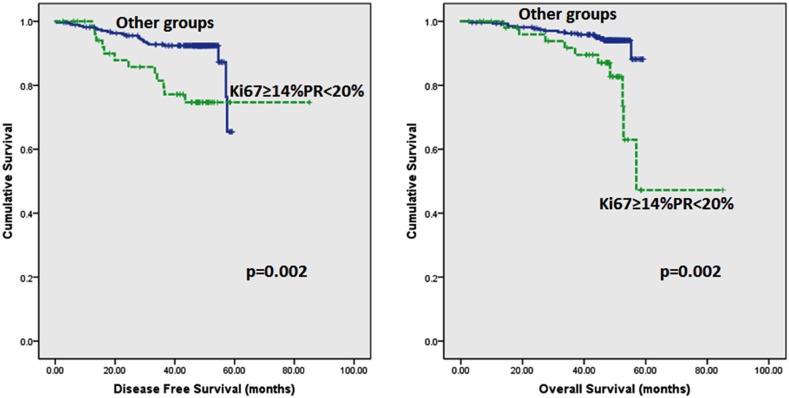
Kaplan-Meier disease free survival and overall survival curves of patients with tumours exhibiting an immunoprofile of Ki67≥14%PR<20% (Luminal B) compared with other immunoprofiles in ER positive tumours only

Follow up of the patients ranged from 2.6 to 62 months (5.2 years) with a mean of 44.8 months and median of 48.2 months. Recurrences occurred in 53 (12.0%) patients in the whole series and 35 (10.2%) in the ER positive series. Breast-cancer specific death was recorded in 42 (9.5%) and 26 (7.6%) women in the whole and ER positive series respectively. On Kaplan–Meier analysis, we found that patients whose tumours harboured Ki67 proliferation rate at 14% and greater disclosed both poorer DFS (p=0.008 and p=0.005) and OS (p=0.006 and p=0.007) (Figure 2 and 3) in the whole series and ER positive series respectively. Additionally, patients with a combinational phenotype of Ki67≥14%PR<20% showed unfavourable DFS (p=0.003 and p=0.002) and OS (p=0.001 and p=0.002) in both the whole series as well as the ER positive series (Figure 4 and 5).

On multivariate analysis (adjusted for age, tumour size, histologic grade and axillary lymph node status), tumours with high proliferation rate (Ki67 ≥14%) and tumours which harboured high proliferation rate accompanied by PR<20%, had increased likelihood of recurrences (HR 1.941, 95%CI 1.105-3.408, p=0.021), (HR 2.069, 95%CI 1.168-3.667, p=0.013) and shorter OS (HR 2.201, 95%CI 1.159-4.180, p=0.016), (HR 2.491, 95%CI 1.309-4.740, p=0.005) respectively in the whole series (Table 6). Similar findings were observed among ER positive cases (Table 7). Of the latter, 105 tumours disclosed ER+HER2-PR≥20% immunoprofile which may correspond to the Luminal A molecular subtype by immunohistochemical surrogate [15]. The value of Ki67 associated with this group of tumours was found to be <17% (Table 8).

**Table 6:** Multivariate analysis on whole series

Whole series	Multivariate analysis on DFS	Multivariate analysis on OS
**Tumours with Ki67≥14%**	p=0.021, HR 1.941	p=0.016, HR 2.201
Yes vs No	(95%CI 1.105-3.408)	(95%CI 1.159-4.180)
**Tumours with PR<20%**	p=0.059, HR1.756	p=0.052 HR 1.942
Yes vs No	(95%CI 0.980-3.149)	(95%CI 0.993-3.797)
**Tumours with Ki67≥14%PR<20%**	p=0.013, HR 2.069	p=0.005, HR 2.491
Yes vs No	(95%CI 1.168-3.667)	(95%CI 1.309-4.740)

**Table 7:** Multivariate analysis on ER positive series

ER+ series	Multivariate analysis on DFS	Multivariate analysis on OS
**Tumours with Ki67≥14%**	p=0.012, HR 2.409	p=0.012, HR 2.787
Yes vs No	(95%CI 1.215-4.775)	(95%CI 1.247-6.231)
**Tumours with PR<20%**	p=0.277, 0.682	p=0.372, HR0.694
Yes vs No	(95%CI 0.342-1.360)	(95%CI 0.311-1.547)
**Tumours with Ki67≥14%PR<20%**	p=0.005, HR 2.896	p=0.005, HR 3.342
Yes vs No	(95%CI 1.388-6.041)	(95%CI 1.441-7.754)

**Table 8:** Ki67 value associated with ER+HER2-PR≥20% tumours

ER+HER2-PR≥20%	Total	Ki67<17%	Ki67≥17%	P value	Pearson correlation
Yes	105	105 (100%)	0 (0%)	<0.001	0.519
No	238	130 (54.6%)	108 (45.4%)		

## DISCUSSION

Gene expression based classification of breast cancers is heavily influenced by genes involved in tumour proliferation. This is of particular significance in ER positive breast cancer which can be stratified into prognostic subgroups primarily on the basis of proliferation. Mitotic activity is a key component of the modified Scarff-Bloom-Richardson system that is now universally used to grade breast cancers. Routine clinical application of Ki67 immunohistochemistry, however, has been fraught with many obstacles even though studies have proven a relationship between Ki67 and survival outcomes [1–3]. An optimal cut-off value of Ki67 that can prognosticate patients into high and low risk groups remains elusive. There is also no universal agreement on the optimal cut-off value to differentiate Luminal B from Luminal A subtypes of breast cancers amongst ER positive, HER2 negative tumours. The reason for this is the many pre-analytical and analytical factors that come into play in assessing Ki-67 immunohistochemistry. These include the type of Ki67 antibody applied [16], type of specimens used to score Ki67 (whole slide, core biopsy or TMA)[17], the area of the tumour selected for scoring [18] and the method of scoring [12][19]. Scoring methods are broadly divided into visual assessment or CAIA methods and there are many different ways of assessment even within each group. Intratumoural heterogeneity further complicates the issue of region selection. In our study, we employed a CAIA platform to perform Ki67 scoring in order to eliminate interobserver variability associated with visual assessment methods. We used the MIB1 antibody clone as this is the most widely applied antibody against Ki67 and we have demonstrated a strong correlation between this antibody and Ki67 mRNA expression in our study. For construction of TMA cores, we selected 3 regions that were representative of the tumour on H&E stained sections. Amongst the 3 cores constructed from each tumour, the core with the highest Ki67 score on CAIA was used for statistical analysis, mirroring how mitotic counts from the most mitotically active area, rather than an average, is used for tumour grading. Our findings showed that ER positive tumours with Ki67 ≥14% had poorer DFS and OS.

Some other studies which also used CAIA platforms have found a similar prognostic cut-off value of 11.5% [20] and 12% [21] with the latter being a large, multicenter study involving more than 8000 breast cancer patients. The difference in cut-off values between those two studies and ours is likely due to variations in the study design. The study by Abubakar et. al.[21]used an average of the Ki67 scores in cases with more than 1 TMA core while the study by Arihiro et. al.[20]analyzed whole tissue sections. Interestingly, both studies showed a higher cut-off value for visual estimation methods which were performed and compared against the CAIA platform. Abubakar et. al. found a visual cut-off value that was optimal at 25% while Arihiro’s finding was 28.5%. This could lend credence to the higher cut-off value of 20% proposed at the 2013 St Gallen consensus meeting [5] as well as the 20% cut-off value proposed by other studies [8, 22–24]. According to one study [16], the difference between the cut-off values obtained by CAIA and visual assessment methods could be due to the generally higher number of tumour cells evaluated by the CAIA platform compared to the human evaluator at the microscope. This larger number of cells helps to reduce the error risk.

Based on the study by Prat et al [15]which found that a PR cut point of ≥20% corresponds more closely to the luminal A subtype of breast cancer, our cohort has 105 tumours with a ER+HER2-PR≥20% (Luminal A) phenotype. We found that the most suitable cut-off value of Ki67 to define this phenotype as opposed to ER+HER2-PR<20% (Luminal B) tumours was <17%. This value is in between the cut-off recommended at the 2011 St Gallen consensus meeting [4] of 14% and the preferred value voted by the majority of panelists at the 2013 St Gallen consensus meeting of 20%[5]. We concede that 17% is a value that is difficult to apply clinically unless quantitative scoring of Ki67 is performed. If visual assessment methods are used, a 20% cut-off value is likely to be more practical. We also found that PR<20% alone does not predict outcome (Tables 6 and 7) However, a combinational phenotype of Ki67≥14% and PR<20% conferred a poorer DFS and OS in this study.

One limitation of our study is that the optimal Ki67 cut-off value that is determined may not be relevant in other laboratories that use a different CAIA platform or method of region selection. While other studies which use different CAIA platforms to quantitate Ki67 have yielded different cut-off values, we found at least two studies that reported a cut-off value that is close to our threshold of 14%[20, 21]. In addition, the strong agreement of analysis results between the Definiens and Aperio platforms on slides scanned using different whole slide scanners shows that our results can be reproduced on at least one other image analysis platform. The use of TMA in our study may mean that our findings cannot be directly extrapolated to routine histopathology service where whole sections are analyzed, although our assessment of 3 TMA cores of 1mm diameter each with the highest Ki67 index for analysis may be considered representative of proliferation assessment of the whole tumour. Additionally, a study by Kobierzycki et. al.[25] involving 51 cases found excellent correlation of Ki67 protein expression between TMAs and whole sections.

In conclusion, through the use of CAIA, we have found that Ki67≥14% in invasive breast cancers confers a poorer DFS and OS on multivariate analysis while Ki67≥17% is more strongly associated with ER+HER2-PR<20% (Luminal B) tumours. The different Ki67 thresholds with regard to prognosis and that associated with definition of luminal B tumours in our study need further rationalization and investigation, and could be related to underlying tumour biology. Given the interobserver variability present in visual assessment methods, CAIA provides an alternative which allows us to determine the Ki67 proliferation index of tumours in a quantitative and reproducible manner. This is especially important in patients with ER positive, node negative breast cancers where the Ki67 proliferative index may influence decisions regarding adjuvant systemic therapy. Given the wide availability of Ki67 immunohistochemistry as well as the modest cost, methods to improve interobserver variation and enhance reproducibility are worthwhile endeavours. We have demonstrated that CAIA is a feasible, reproducible and quantitative method for determination of a Ki67 proliferative index, with a strong correlation with breast cancer outcome. Further investigation of this method is therefore warranted to improve standardization of methodology and applicability in routine clinical practice.

## MATERIALS AND METHODS

### Patients and tumours

The study cohort is comprised of 440 patients with invasive breast cancer diagnosed in 2012 at the Department of Anatomical Pathology, Singapore General Hospital.

### Tissue microarray (TMA) construction

Histological slides were retrieved and reviewed. Representative areas were selected and tissue microarrays were constructed using Beecher Microarrayer with three 1mm cores constructed from each case.

### Immunohistochemistry

Immunohistochemistry was performed on tissue microarray sections using antibody to Ki67 (MIB1 clone; Dako M 7240; dilution 1:100). Sections (4μm thick) were cut from the TMA blocks, mounted on Leica Microsystems Plus slides and dried on a heating bench for 20minutes. The immunohistochemical staining procedure was performed using the Leica Bond Autostainer (Leica Biosystem, Newcastle Ltd, UK). The slides were placed on Bond trays and covered with cover tiles and loaded into the system. The sections were deparaffinised and pretreated using bond dewax reagents and ER2 antigen retrieval buffer of pH 8.9 to 9.1. Endogenous peroxidase activity was blocked using hydrogen peroxide for 5 minutes followed by primary antibody incubation for 20minutes. The sections were then treated with post primary and polymer reagents followed by a mixed DAB refine reagent. The detection system used was Bond polymer refine detection (DS9800). The sections were counterstained with haematoxylin and the slides were unloaded from the system, dehydrated and mounted in depex mounting medium. ER, PR and HER2 status was recorded from histological reports. In our laboratory, the SP1 clone (Neomarker RM9101-S; dilution 1:50) was used for ER immunohistochemistry, PgR636 clone (Neomarker RM9102-S; dilution 1:200) was used for PR while the SP3 clone (Neomarker RM9103-S; dilution 1:200) was used for HER2. For ER and PR immunohistochemistry, a result was considered positive if at least 1% of the lesional cells displayed any intensity of unequivocal nuclear staining. For HER2, a test was considered positive if more than 10% of the lesional cells exhibited 3+ cell membrane staining.

### Quantitative immunoscoring using computer assisted image analysis (CAIA)

**Figure 6:**
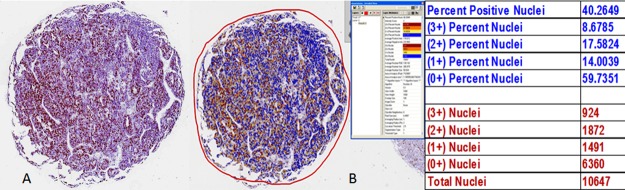
Determination of Ki67 analysis by the Aperio ePathology Immunohistochemistry Nuclear Image Analysis algorithm

Ki67 immunoreactivity was determined by the Aperio ePathology ImmunoHistochemistry Nuclear Image Analysis algorithm on slides scanned via Aperio ScanScope XT System using 20x equivalent objective. Prior to running the algorithm, three pathologists (NP, ARJ and JI) used the ImageScope annotation tools to outline the tumour-cell only regions to manually delineate these from stroma, inflammatory cells, necrosis and other non-tumour or non-viable regions within each TMA core. The concurrence of the 3 pathologists ensured that only the tumour cells would be subjected to image analysis. The IHC Nuclear Image Analysis algorithm detected the nuclear staining for a target chromogen for the individual cells in those regions and quantified the intensity. Nuclear staining was classified as 0 (nil), 1+ (weak staining), 2+ (moderate staining), and 3+ (strong staining) based on staining intensity and the percentage of each staining intensity was recorded (Figure 6). The Ki67 score was derived from the sum total of the percentages of different staining intensity. Ki67 positive lymphocytic infiltrates were excluded from the analysis algorithm.

All 3 TMA cores of each case were subjected to the Ki67 quantitative analysis. However, only the core which yielded the highest Ki67 proliferation rate was selected for analysis. For validation of the Ki67 quantitative analysis by Aperio, 52 cases were subjected to Ki67 quantitative analysis using Definiens Tissue Studio (version 4.4) on slides scanned via Philips Intellisite Ultra Fast Scanner using 40x equivalent objective (0.25 μm/pixel) and stored on Philips Image Management System (version 2.4).

### RNA extraction, NanoString gene expression analysis

Among the study cohort, 37 cases were also subjected to Ki67 gene expression analysis. 10μm unstained standard sections of the selected paraffin tumour blocks were subjected to RNA extraction using the RNeasy FFPE kit (Qiagen, Hilden, Germany) on a QIAcube automated sample preparation system (Qiagen, Hilden, Germany) and was quantified by an Agilent 2100 Bioanalyzer system (Agilent, Santa Clara, CA, USA). 100ng of functional RNA (>300 nucleotides) was assayed on an nCounter Custom CodeSet (NanoString Technologies, Seattle, WA, USA). NanoString counts were normalized using the positive control probes as well as the housekeeping genes.

### Follow up data

Follow-up data was obtained from patient clinical case notes. Disease-free survival (DFS) and overall survival (OS) were defined as time from diagnosis to recurrence (local or systemic) or death/date of last follow up respectively. There were no follow up data in 15 (3.4%) women in the whole series and 13 (3.8%) in the ER positive cases. Patients with no follow up data were excluded from the survival analysis.

### Statistical analysis

The findings were analyzed using the statistical software SPSS for Windows, Version 21. The correlation between Ki67 mRNA expression and Ki67 IHC was evaluated by Spearman’s rank correlation. Linear regression was also performed to evaluate the relationship between these two parameters. Survival outcomes were estimated with the Kaplan–Meier analysis using various cutoff values of Ki67 immunoreactivity such as 10%, 12%, 14%, 15% and 20% to assess for significance and compared between groups (as shown in Tables 6 and 7) with the log-rank statistics. Cox proportional hazards models were used to determine the effect of combinational phenotypes on survival outcomes. Hazard ratios together with 95% confidence intervals were reported for the outcomes and a p-value of 0.05 defined statistical significance. In assessing the level of agreement between the Aperio (on slides scanned using Aperio solution) and Definiens solution (on slides scanned using Philips solution), the kappa statistic for categorical variables using the statistically significant Ki67 cutoff value of 14% (see Results), and intraclass correlation coefficient (ICC) for continuous variables, were used. Values of kappa from 0 to 0.2 were regarded as indicating no agreement, 0.21–0.4 fair agreement, 0.41–0.6 moderate agreement, 0.61–0.8 substantial agreement, and 0.81–1 almost perfect agreement. The intraclass correlation coefficient is a general measurement of agreement or consensus for parametric measurements, with values of 0–0.2 indicating poor agreement, 0.3–0.4 fair agreement, 0.5–0.6 moderate agreement, 0.7–0.8 strong agreement, and >0.8 almost perfect agreement. For comparison of the tumour cell count across the 3 TMA cores as well as between the Aperio and Definiens system, we used the intraclass correlation coefficient.

## References

[1] Bouzubar N, Walker KJ, Griffiths K, Ellis IO, Elston CW, Robertson JF, Blamey RW, Nicholson RI (1989). Ki67 immunostaining in primary breast cancer: pathological and clinical associations. Br J Cancer.

[2] Sahin AA, Ro JY, El-Naggar AK, Ordonez NG, Ayala AG, Ro J, Hortobagyi GN, Blick MB, Fritsche HA, Smith TL (1991). Ki-67 immunostaining in node-negative stage I/II breast carcinoma. Significant correlation with prognosis. Cancer.

[3] Munzone E, Botteri E, Sciandivasci A, Curigliano G, Nolè F, Mastropasqua M, Rotmensz N, Colleoni M, Esposito A, Adamoli L, Luini A, Goldhirsch A, Viale G (2012). Prognostic value of Ki-67 labeling index in patients with node-negative, triple-negative breast cancer. Breast Cancer Res Treat.

[4] Goldhirsch A, Wood WC, Coates AS, Gelber RD, Thürlimann B, Senn HJ (2011). Strategies for subtypes-dealing with the diversity of breast cancer: Highlights of the St Gallen international expert consensus on the primary therapy of early breast cancer 2011. Ann Oncol.

[5] Goldhirsch A, Winer EP, Coates AS, Gelber RD, Piccart-Gebhart M, Thürlimann B, Senn HJ, Albain KS, André F, Bergh J, Bonnefoi H, Bretel-Morales D, Burstein H (2013). Personalizing the treatment of women with early breast cancer: Highlights of the st gallen international expert consensus on the primary therapy of early breast Cancer 2013. Ann Oncol.

[6] Aleskandarany MA, Green AR, Benhasouna AA, Barros FF, Neal K, Reis-Filho JS, Ellis IO, Rakha EA (2012). Prognostic value of proliferation assay in the luminal, HER2-positive, and triple-negative biologic classes of breast cancer. Breast Cancer Res.

[7] Kurozumi S, Matsumoto H, Hayashi Y, Tozuka K, Inoue K, Horiguchi J, Takeyoshi I, Oyama T, Kurosumi M (2017). Power of PgR expression as a prognostic factor for ER-positive/HER2-negative breast cancer patients at intermediate risk classified by the Ki67 labeling index. BMC Cancer.

[8] Rossi L, Laas E, Mallon P, Vincent-Salomon A, Guinebretiere JM, Lerebours F, Rouzier R, Pierga JY, Reyal F (2015). Prognostic impact of discrepant Ki67 and mitotic index on hormone receptor-positive, HER2-negative breast carcinoma. Br J Cancer.

[9] Inwald EC, Klinkhammer-Schalke M, Hofstädter F, Zeman F, Koller M, Gerstenhauer M, Ortmann O (2013). Ki-67 is a prognostic parameter in breast cancer patients: Results of a large population-based cohort of a cancer registry. Breast Cancer Res Treat.

[10] Kashiwagi S, Yashiro M, Takashima T, Aomatsu N, Ikeda K, Ogawa Y, Ishikawa T, Hirakawa K (2011). Advantages of adjuvant chemotherapy for patients with triple-negative breast cancer at Stage II: usefulness of prognostic markers E-cadherin and Ki67. Breast Cancer Res.

[11] Mengel M, Von Wasielewski R, Wiese B, Rüdiger T, Müller-Hermelink HK, Kreipe H (2002). Inter-laboratory and inter-observer reproductibility of immunohistochemical assessment of the Ki-67 labelling index in a large multi-centre trial. J Pathol.

[12] Gudlaugsson E, Skaland I, Janssen EA, Smaaland R, Shao Z, Malpica A, Voorhorst F, Baak JPA (2012). Comparison of the effect of different techniques for measurement of Ki67 proliferation on reproducibility and prognosis prediction accuracy in breast cancer. Histopathology.

[13] Laurinavicius A, Plancoulaine B, Laurinaviciene A, Herlin P, Meskauskas R, Baltrusaityte I, Besusparis J, Dasevicius D, Elie N, Iqbal Y, Bor C (2014). A methodology to ensure and improve accuracy of Ki67 labelling index estimation by automated digital image analysis in breast cancer tissue. Breast Cancer Res.

[14] Stålhammar G, Fuentes Martinez N, Lippert M, Tobin NP, Mølholm I, Kis L, Rosin G, Rantalainen M, Pedersen L, Bergh J, Grunkin M, Hartman J (2016). Digital image analysis outperforms manual biomarker assessment in breast cancer. Mod Pathol.

[15] Prat A, Cheang MC, Martín M, Parker JS, Carrasco E, Caballero R, Tyldesley S, Gelmon K, Bernard PS, Nielsen TO, Perou CM (2013). Prognostic significance of progesterone receptor-positive tumour cells within immunohistochemically defined luminal a breast cancer. J Clin Oncol.

[16] Fasanella S, Leonardi E, Cantaloni C, Eccher C, Bazzanella I, Aldovini D, Bragantini E, Morelli L, Cuorvo LV, Ferro A, Gasperetti F, Berlanda G, Dalla Palma P, Barbareschi M (2011). Proliferative activity in human breast cancer: Ki-67 automated evaluation and the influence of different Ki-67 equivalent antibodies. Diagn Pathol.

[17] Knutsvik G, Stefansson IM, Aziz S, Arnes J, Eide J, Collett K, Akslen LA (2014). Evaluation of Ki67 expression across distinct categories of breast cancer specimens: A Population-based study of matched surgical specimens, core needle biopsies and tissue microarrays. PLoS One.

[18] Christgen M, Von Ahsen S, Christgen H, Länger F, Kreipe H (2015). The region-of-interest size impacts on Ki67 quantification by computer-assisted image analysis in breast cancer. Hum Pathol.

[19] Focke CM, van Diest PJ, Decker T (2016). St Gallen 2015 subtyping of luminal breast cancers: impact of different Ki67-based proliferation assessment methods. Breast Cancer Res Treat.

[20] Arihiro K, Oda M, Ohara M, Kadoya T, Osaki A, Nishisaka T, Shiroma N, Kobayashi Y (2016). Comparison of visual assessment and image analysis in the evaluation of Ki-67 expression and their prognostic significance in immunohistochemically defined luminal breast carcinoma. Jpn J Clin Oncol.

[21] Abubakar M, Orr N, Daley F, Coulson P, Ali HR, Blows F, Benitez J, Milne R, Brenner H, Stegmaier C, Mannermaa A, Chang-Claude J, Rudolph A (2016). Prognostic value of automated KI67 scoring in breast cancer: a centralised evaluation of 8088 patients from 10 study groups. Breast Cancer Res.

[22] Stathopoulos GP, Malamos NA, Markopoulos C, Polychronis A, Armakolas A, Rigatos S, Yannopoulou A, Kaparelou M, Antoniou P (2014). The role of Ki-67 in the proliferation and prognosis of breast cancer molecular classification subtypes. Anticancer Drugs.

[23] Tashima R, Nishimura R, Osako T, Nishiyama Y, Okumura Y, Nakano M, Fujisue M, Toyozumi Y, Arima N (2015). Evaluation of an optimal cut-off point for the Ki-67 index as a prognostic factor in primary breast cancer: A retrospective study. PLoS One.

[24] Bustreo S, Osella-Abate S, Cassoni P, Donadio M, Airoldi M, Pedani F, Papotti M, Sapino A, Castellano I (2016). Optimal Ki67 cut-off for luminal breast cancer prognostic evaluation: a large case series study with a long-term follow-up. Breast Cancer Res Treat.

[25] Kobierzycki C, Pula B, Wojnar A (2012). Tissue Microarray Technique in Evaluation of Proliferative Activity in Invasive Ductal Breast Cancer. Anticancer Res.

